# Research on the influencing factors of the development of metabolic syndrome in patients with schizophrenia and the intervention effect of dance movement therapy

**DOI:** 10.3389/fpsyt.2025.1676286

**Published:** 2025-12-10

**Authors:** Suhong Wang, Yue Zhou, Xueli Zhu, Yangyang Yu, Weixia Xiao, Qianqian Sun, Fang Yan, Chuansheng Wang

**Affiliations:** 1 Department of Psychiatry VI, The Second Affiliated Hospital of Xinxiang Medical University (Henan Mental Hospital), Xinxiang, China; 2 The Second Affiliated Hospital of Xinxiang Medical University (Henan Mental Hospital), Xinxiang, China

**Keywords:** schizophrenia, metabolic syndrome, influencing factors, dance movement therapy, psychosocial intervention, intervention effect, inflammation

## Abstract

**Objectives:**

Patients with schizophrenia are at increased risk for metabolic syndrome (MS), which can lead to cardiovascular diseases and diabetes, potentially worsening mental symptoms and hindering recovery. Dance movement therapy (DMT), a non-pharmacological intervention that combined movement with psychological regulation, may improve mood and cognition. This study aimed to investigate factors influencing MS in schizophrenia and evaluate the intervention effect of DMT.

**Methods:**

A total of 160 patients with schizophrenia who were admitted to our hospital from April 2021 to April 2023 were prospectively included. According to whether MS occurred, patients were allocated into the MS group (n=80) and the non-MS group (n=80). Logistic regression was used to analyze factors influencing MS. Subsequently, the MS group was randomly allocated to a control group (n=40), which received conventional treatment, and an intervention group (n=40), which received DMT in addition to conventional care. The metabolic indicators and positive and negative syndrome scale (PANSS) scores of both groups before and after the intervention were collected to evaluate the intervention effect of DMT.

**Results:**

The advanced age, smoking habit, high BMI and interleukin-6 (IL-6) levels, long medication time, and elevated CRP levels were risk factors for the development of MS. Moderate exercise habits were protective factors. After the DMT intervention, body mass index (BMI), waist circumference (WC), diastolic blood pressure (DBP), and PANSS scores of the intervention group showed a downward trend as the intervention time increased. However, the BMI, WC, DBP, and PANSS scores of the control group displayed an increasing tendency. In addition, the BMI, WC, DBP, fasting blood glucose (FBG), and PANSS scores of the intervention group were less than those of the control group 4 weeks and 8 weeks after the intervention.

**Conclusion:**

Age, smoking habit, BMI and IL-6 levels, medication time, elevated CRP levels, and exercise habit were the influencing factors for the development of MS in schizophrenia. DMT could effectively improve metabolic parameters and psychiatric symptoms in these patients. However, the study has limitations such as a short intervention period and a single-center design. Future research should verify this results through multi-center studies and longer follow-up periods.

## 1 Introduction

Schizophrenia is a serious chronic mental disorder, characterized mainly by scattered associations, logical confusion, auditory hallucinations that are critical or commanding, decline in working memory, attention, emotional blunting or inappropriate responses, diminished will, social withdrawal, and abnormal behavior (1). The global prevalence rate of this disease is approximately 1% (2, 3). The prevalence rate of schizophrenia among men is notably exceeding that among women, with a gender-specific risk ratio of 1.4:1 (4). The studies have shown that sex hormones can affect the probability of metabolic syndrome (MS) in patients with schizophrenia by regulating the level of interleukin (IL)-1β C-511T in the body (5). Moreover, this disease requires long-term medication treatment. But excessive use of common antipsychotic drugs (especially atypical antipsychotic drugs) may cause metabolic disorders and further increase the probability of developing MS (6).

MS was characterized by central obesity, abnormal blood glucose, dyslipidemia, and hypertension, which can exacerbate the core cognitive impairments in patients with schizophrenia through mechanisms such as insulin resistance and chronic inflammation (7). A study found that the cognitive function was negatively correlated with the level of tumor necrosis factor-α (TNF-α) (8). Moreover, the prevalence of MS in patients with schizophrenia ranged from 16.5% to 43.9%, which is approximately 2 to 3 times that of the general population (9). The situation is particularly severe among hospitalized patients with schizophrenia, where the prevalence rises to between 30.7% to 45.09% (10). This is because the cognitive impairment of patients with schizophrenia leads to the formation of unhealthy habits, thereby further promoting the occurrence of MS (11). The development of MS may increase the risk of cardiovascular diseases and diabetes, impair cognitive function, reduce medication adherence, raise the rate of disease recurrence, and decrease overall quality of life (12). The main reasons for the development of MS may involve internal factors of the disease, drug factors, lifestyle, social psychological factors, and the levels of inflammatory factors (13, 14). Therefore, it is crucial to elucidate the influencing factors of its development, screen high-risk populations, and conduct early intervention.

To reduce the risk of the occurrence of MS, drug adjustment, lifestyle intervention, regular monitoring, and non-drug intervention are mainly used in clinical practice (15). Recent studies have shown that exercise intervention is an effective non-drug treatment method, which can significantly improve the positive, general, and total symptoms, cognitive ability, working memory, social cognition, and attention of patients with schizophrenia (16–18). Moreover, functional mobility is not only an important predictor of cognitive ability and quality of life in patients with schizophrenia, but also plays a regulatory role in the process of MS affecting cognition and quality of life (19). Among them, Dance movement therapy (DMT), as an exercise intervention method combining physical movement, psychological expression, and social interaction, has demonstrated unique value in improving metabolic parameters and mental symptoms of patients with schizophrenia (20). DMT belongs to creative arts therapy, which regulates various pathological physiological processes of schizophrenia patients through core components such as structured group exercises, rhythm-synchronized activities, and interpersonal movement interactions. The regular group aerobic exercise component can effectively improve glucose and lipid metabolism, lower inflammatory marker levels, and regulate the hypothalamic-pituitary-adrenal (HPA) axis function, thereby alleviating metabolic abnormalities and stress-related symptoms. At the same time, the non-verbal expression and interpersonal movement interactions components in DMT can promote sensory integration and the activation of mirror neuron systems, thereby improving emotional recognition ability, reducing negative symptoms (such as social withdrawal), and enhancing social cognitive functions (21). This therapy does not require a background in dance, and participants mobilize their body memories to release inner emotions, thereby achieving the treatment (21). Currently, DMT is used in most studies focusing on neuropsychiatric disorders (22). Its effect on metabolic disorders in patients with schizophrenia still needs further exploration.

Therefore, this study systematically explored the influencing factors of the development of MS in schizophrenia and assessed the function of the DMT treatment as a non-pharmaceutical intervention on improving the metabolic indicators and psychological symptoms of patients. The aim was to provide a basis for formulating prevention and treatment strategies for MS in schizophrenia, thereby improving patients’ cognitive function, enhancing their quality of life, and ultimately reducing the burden of social medical expenses.

## 2 Materials and methods

### 2.1 Patient population

This study prospectively included 160 patients with schizophrenia. These patients were collected at our hospital from April 2021 to April 2023, including 80 patients with schizophrenia accompanied by MS (MS group) and 80 patients with schizophrenia without MS (non-MS group), which met the inclusion requirements of the minimum sample size. Schizophrenia was diagnosed according to the fifth edition of the Diagnostic and Statistical Manual of Mental Disorders (DSM-5) (23). MS was diagnosed according to the 10th edition of the International Diabetes Federation (IDF) (24). Written informed consent was obtained from all participants, and the study protocol was approved by the Ethics Committee of our hospital (Approval Number: XYEFYLL 2025-74).

### 2.2 Randomization and blinding

**Figure 1:**
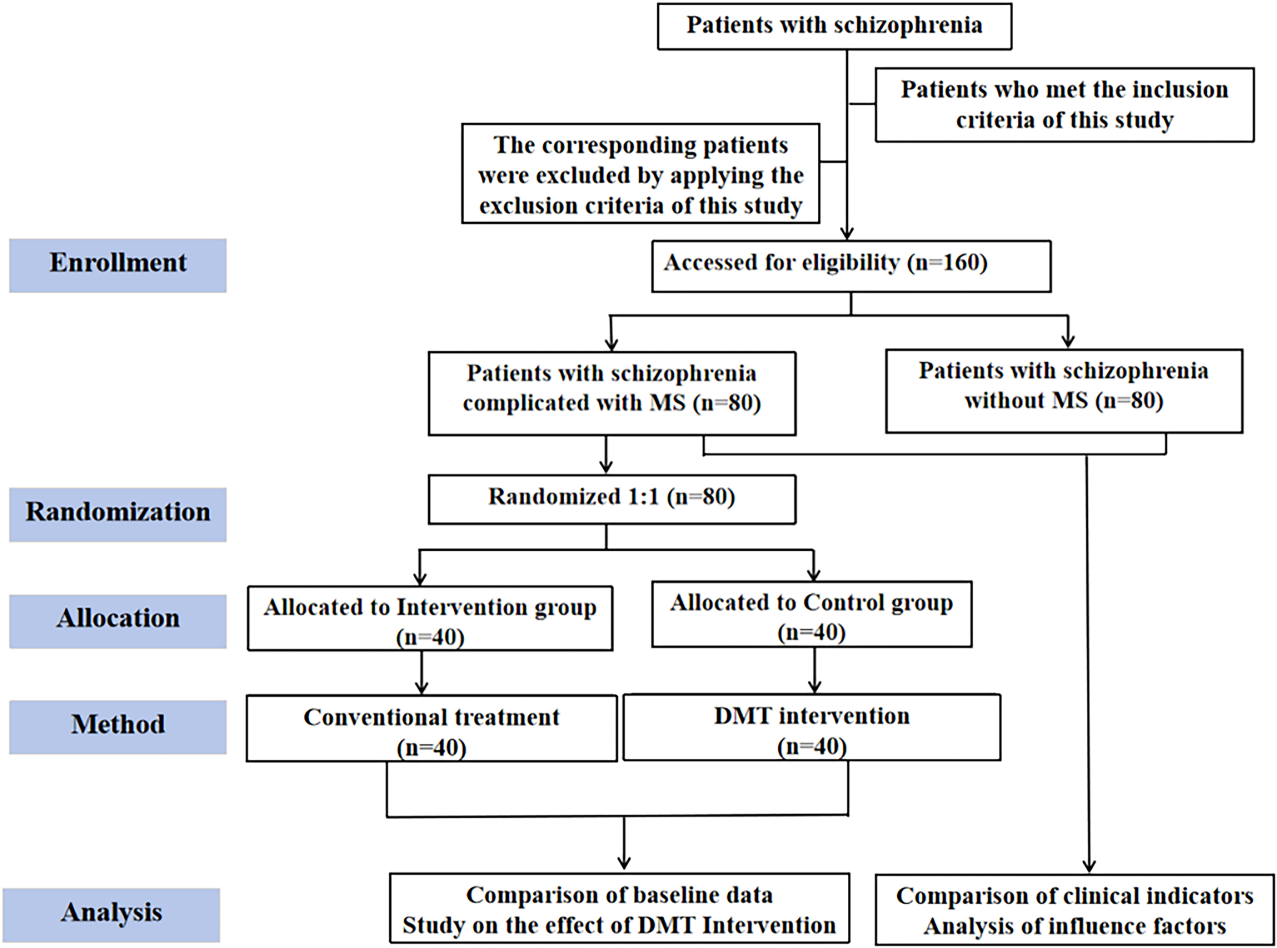
Research design flowchart.

All eligible MS patients were randomly assigned to the experimental group (n=40) or the control group (n=40) in a 1:1 ratio. The randomization sequence was generated by an independent statistician who was not involved in the recruitment, intervention, or outcome assessment of the study. The sequence was generated using the block randomization method, with group sizes of 4 and 6 randomly mixed to maintain the balance of sample sizes between the groups. To ensure the concealment of the allocation, the randomization information was sealed in a series of sequentially numbered, opaque envelopes. When patients completed the informed consent and all baseline assessments, the research coordinator opened the envelopes in sequence to determine their group allocation. Patients in the control group only received conventional treatment, while patients in the intervention group received DMT treatment in addition to the conventional treatment (**Figure 1**). The intervention period lasted for 8 weeks. This duration was determined based on previous research evidence (20), which indicated that this period was sufficient to preliminarily observe the regulatory effect of DMT on metabolic indicators and psychological symptoms, while also taking into account the feasibility of clinical practice and the expected compliance of patients. This study strictly followed the principle of blinding: all participants were unaware of their group assignment and the treatment they received. Due to the nature of the intervention measures, although the rehabilitation therapists were aware of the actual treatment allocation, they strictly kept it confidential throughout and did not participate in the result evaluation; all outcome indicators were collected and evaluated by research assistants who were completely unaware of the group assignments, thus ensuring the objectivity of the assessment process. This blinding setup was maintained from the start of the intervention until the collection and analysis of all data were completed.

### 2.3 Inclusion, exclusion, dropout, and withdrawal criteria

Inclusion Criteria: The patients were compliant with the diagnostic criteria for schizophrenia. Patients without MS were assigned to the non-MS group, while patients who were compliant with the MS diagnostic criteria were assigned to the MS group; The age ranges from 18 to 60 years old; Patients who have a certain ability of mobility and being able to adapt some physical labor; Patients who have a certain ability of oral expression; Patients who cooperate with the progress of the study; Both the patient and his/her family gave informed consent.

Exclusion Criteria: Patients with severe suicidal attempts or impulsive behaviors; Patients with severe intellectual disability or serious physical illness; Patients with difficulties in oral expression or movement; Patients with recent use of opioid medications; Patients who have received electroconvulsive therapy without convulsions or repetitive transcranial magnetic stimulation.

Dropout Criteria and Withdrawal Criteria: Patients who have lost contact for some reason; Patients who voluntarily withdraw from the trial; Patients who also participated in other trial studies during this research period; Patients who reported physical discomfort during the research process.

### 2.4 Research methods

#### 2.4.1 Collection of general information

General information of hospitalized schizophrenia patients was collected through self-made questionnaires, including gender, age, body mass index (BMI), educational level, marital situation, smoking habit (a cumulative continuous smoking period of at least six months and a smoking cessation period of less than five months is considered smoking), drinking alcohol situation (2 bottles of beer per time or 50 mL of high-concentration liquor per time, more than 5 times a week, and a duration of no less than 1 hour is considered drinking), the family history of MS (a family history of any one of hypertension, diabetes, or hyperlipidemia), number of hospitalizations, age of onset, medication time, systolic blood pressure (SBP), diastolic blood pressure (DBP), and exercise habits (the standard for moderate exercise is 20 to 25 min/d at high intensity or 30~60 min/d at moderate intensity, for 3 to 5 days a weeks), sleep disorder situation (Pittsburgh Sleep Quality Scale index score≥8 points) (25), depression situation (Zung Depression Scale score≥53 points) (26). The levels of plasma interleukin-6 (IL-6) and plasma high-sensitivity C-reactive protein (hs-CRP) of the patients were collected. A level of hs-CRP≥3 mg/L was considered an elevated CRP level. The mental status of patients was evaluated by using the Positive and Negative Syndrome Scale (PANSS) (27).

#### 2.4.2 Design and implementation

The hospitalized MS group patients were randomly allocated into the control group and the intervention group. The control group received conventional treatment, while the intervention group was administered the DMT treatment in addition to the conventional treatment. The course content was designed and implemented by mental rehabilitation therapists (authorized by the hospital’s clinical application medical technology management committee). DMT was divided into four stages, each consisting of different courses. Each course lasted 90 minutes, 3 times per week, and the treatment lasted for 8 weeks. The specific design of the DMT course was as follows: I. Stages 1 to 4: Group establishment stage. Help members understand each other and break down emotional barriers through the form and rules of group activities. Gradually establish connections. The contents of the activity included: 1) Dance movement psychological education; 2) Introduce the group setup, safety rules and instructions; 3) Warm-up activities and breathing training, perceiving the body; 4) Theme activities: interactive circle formation, choreographed name dance, creative greeting movements, rhythmic movements, closing dance; 5) Write down your feelings anonymously and share them by drawing lots. II. Stages 5 to 10: Experience and Creation Stage. Through the contrast and expression of actions, suppressed emotions are released, the body and mind are integrated, creativity is stimulated, and the trust and connection ability with reality among members are enhanced. The contents of the activity included: 1) Warm-up dance (greeting dance); 2) Theme activities: action mirroring, perception and recognition, strengthening physical strength, emotions, center of gravity, boundaries, space, size, orientation, and elasticity, imagery expression of vocalization, rhythmic movements, and conclusion of the dance; 3) Sharing session. III. Stages 11 to 20: Interaction and Development Stage. Through sincere motor expression, stimulate kinesthetic awareness, experience the joy of cooperation and creation, help members improve withdrawal behavior, and explore conflict handling and problem-solving abilities through physical interaction. The contents of the activity included: 1) Warm-up dance (body sculpture); 2) Theme activities: group co-creation of action metaphors, such as childhood games, zoo adventures, hunting, planting, harvest, festival celebrations, skits (such as confinement, discharge, epidemic), use ropes to explore relationships, experience tension and relaxation; 3) Sharing session. IV. Stages 21 to 24: Reconstruction and Separation phases. Implant hope, internalize positive images, and promote recovery. The contents of the activity included: 1) Warm-up dance (greeting dance); 2) Theme activities: expressing love and blessings through creative movements, expanding the garden of the soul, and playing games of metal, wood, water, fire, and earth, experience separation and aggregation in action, end the dance; 3) Share feelings and say goodbye.

#### 2.4.3 Collection of therapeutic effect evaluation indicators

By collecting indicators such as BMI, waist circumference (WC), SBP, DBP, fasting blood glucose (FBG), total cholesterol (TC), low-density lipoprotein cholesterol (LDL-C), TG, and PANSS scores of patients before treatment and after treatment (4 weeks and 8 weeks). The therapeutic effect was evaluated by the above indicators. BMI measurement: The patient stood barefoot in the center of the electronic scale, and the weight (kg) was recorded. WC measurement: The patient stood naturally, with feet apart 25–30 cm. At the end of exhalation and before inhalation, use an elastic tape measure to wrap around the waist horizontally at a point 0.5–1 cm above the navel, and read the value accurately to the unit of centimeter (cm). Blood pressure measurement: The patient exposed the right arm, measured, and recorded the SBP and DBP (mmHg) according to the standard method. FBG and blood lipids (TC, LDL-C, TG) were detected by the automatic biochemical analyzer (Beckman AU5800). Patients fasted from 8 p.m. the previous night and had an empty fasting venous blood drawn at 6 a.m. the next morning. The PANSS score (27) was used to assess the severity of the patient’s mental disorder. The higher the score, the more severe the patient’s mental illness.

### 2.5 Observation indicators

The clinical indicators of the MS group and the non-MS group were compared: gender, age, BMI, educational level, marital situation, smoking situation, drinking situation, family history of MS, number of hospitalizations, age of onset, medication time, blood pressure, exercise habits, sleep disorder situation, depression situation, IL-6, elevated CRP levels situation, and total PANSS scores.

The influencing factors of MS in patients with schizophrenia were analyzed: Age, BMI, smoking, IL-6, medication time, exercise habits, and elevated CRP levels.

The clinical indicators of the intervention group and the control group were compared: gender, age, BMI, height, educational level, marital situation, smoking situation, drinking situation, family history of MS, number of hospitalizations, age of onset, medication time, blood pressure, exercise habits, sleep disorder situation, depression situation, IL-6, elevated CRP levels situation, and PANSS scores.

The changes of efficacy evaluation indicators of the intervention group and the control group before treatment and after treatment (4 weeks and 8 weeks) were compared: BMI, WC, SBP, DBP, FBG, TC, LDL-C, TG, and PANSS scores.

### 2.6 Statistical analysis

Based on the assumptions of the cross-sectional study, the PASS 15.0 software was used to calculate the minimum sample size. The test power was set at 90% (1-β=0.90), and the significance level was 0.05 (α=0.05, two-sided test). Based on previous literature (11), the probability of MS occurring in patients with schizophrenia is approximately 34.63%. The minimum sample size was calculated to be 102 patients. There were at least 51 patients in the MS group and at least 51 patients in the non-MS group. Accounting for a 20% attrition rate, the study required a total of 124 participants. In the cross-sectional survey, we will expand the screening sample size to ensure that at least 51 MS patients and 51 non-MS patients can complete all assessments. SPSS 27.0 software was employed for the analysis of the data. To control potential biases and confounding factors, this study employed multiple imputation methods to handle the missing data that might exist. The data’s normality was assessed by the Shapiro-Wilk (SW) test. The mean ± standard deviation (x ± s) was applied to express the measurement data that fit the normal distribution. The independent sample t-test was employed for pairwise comparisons between groups, and the ANOVA approach was employed for comparisons between the three groups. Non-parametric tests were employed for group comparisons, and non-normally distributed data were represented as *M* (*Q_25_*, *Q_75_*). The count data were presented as n (%), and the *χ^2^* test was used to compare the count data groupings. Patients with schizophrenia were screened for MS impacting variables using logistic regression analysis. For the repeated continuous measurement data, a two-way repeated measures analysis of variance was employed to separately evaluate the time effect of the dry TDM intervention (before intervention, 4 weeks of intervention, 8 weeks of intervention), the group effect (intervention group and control group), and the interaction effect (time×group). Statistical significance was defined as a *P* value less than 0.05. To facilitate the comparative analysis, the partial Eta square effect sizes (η²) of the variance analysis were calculated, and the 95% confidence interval (*CI*) of the effect value was reported.

## 3 Results

### 3.1 Clinical indicators comparison between the MS group and the non-MS group

The clinical indicators of the two groups of patients were displayed in **Table 1**. The MS group and the non-MS group showed statistically significant differences in gender, educational level, marital situation, drinking alcohol situation, the family history of MS, number of hospitalizations, age of onset, SBP, DBP, sleep disorder situation, depression situation, and PANSS scores. There were statistically significant differences in age (*t* = 2.602, *p* = 0.010), BIM (*t* = 7.920, *p*<0.001), smoking situation (*χ^2^* = 17.071, *p*<0.001), medication time (*t* = 4.017, *p*<0.001), IL-6 (*t* = 10.197, *p*<0.001), exercise habits (*χ^2^* = 2.602, *p* = 0.020), and elevated CRP levels (*χ^2^* = 4.838, *p* = 0.028).

**Table 1:** Comparison of clinical data between the MS group and the non-MS group.

Indicators	MS group (*N* = 80)	Non-MS group (*N* = 80)	*t/χ^2^*	*P*
	Mean ± SD/n(%)	Mean ± SD/n(%)		
Gender, n(%)			0.914	0.339
Male	42 (52.50)	48 (60.00)		
Female	38 (47.50)	32 (40.00)		
Age, year (mean ± SD)	47.51 ± 9.31	43.39 ± 10.67	2.602	0.010
BMI, kg/m^2^ (mean ± SD)	27.35 ± 2.54	23.57 ± 3.43	7.920	<0.001
Educational level, n(%)			0.625	0.429
High school and below	43 (53.75)	38 (47.50)		
College degree or above	37 (46.25%)	42 (52.50)		
Marital situation, n(%)			0.110	0.740
Married	53 (66.25)	51 (63.75)		
Unmarried	27 (33.75)	29 (36.25)		
Smoking habit, n(%)			17.071	<0.001
Yes	57 (71.25)	31 (38.75)		
No	23 (28.75)	49 (61.25)		
Drinking alcohol, n(%)			0.517	0.472
Yes	61 (76.25)	57 (71.25)		
No	19 (23.75)	23 (28.75)		
The family history of MS, n(%)			1.039	0.595
Hypertension	5 (6.25)	3 (3.75)		
Diabetes	8 (10.00)	7 (8.75)		
Hyperlipidemia	6 (7.50)	2 (2.50)		
Number of hospitalizations, nos (mean ± SD)	6.27 ± 1.57	5.86 ± 1.69	1.589	0.114
Age of onset, year (mean ± SD)	27.07 ± 7.98	28.75 ± 8.89	-1.258	0.210
Medication time, week (mean ± SD)	14.57 ± 3.32	12.32 ± 3.75	4.017	<0.001
IL-6, ng/L (mean ± SD)	2.35 ± 0.32	1.72 ± 0.45	10.197	<0.001
SBP, mm Hg (mean ± SD)	126.97 ± 17.59	125.54 ± 15.05	0.553	0.581
DBP, mm Hg (mean ± SD)	75.32 ± 9.37	74.54 ± 10.05	0.508	0.612
Exercise habit, n(%)			10.503	0.020
Yes	7 (8.75)	23 (28.75)		
No	73 (91.25)	57 (71.25)		
Sleep disorder, n(%)			0.101	0.751
Yes	35 (43.75)	37 (46.25)		
No	45 (56.25)	43 (53.75)		
Depression, n(%)			0.196	0.658
Yes	11 (13.75)	13 (16.25)		
No	69 (86.25)	67 (83.75)		
Elevated CRP levels, n(%)			4.838	0.028
Yes	14 (17.50)	5 (6.25)		
No	66 (82.50)	75 (93.75)		
PANSS score, points (mean ± SD)	73.32 ± 27.46	79.27 ± 23.45	-1.474	0.143

MS, metabolic syndrome; IL-6, interleukin-6; SBP, systolic blood pressure; DBP, diastolic blood pressure; CRP, C-reactive protein.

### 3.2 Influencing factors of the development of MS

Before conducting the Logistic regression analysis, to prevent the occurrence of biased results due to the high correlation among the independent variables, multiple collinearity tests were performed on age, BMI, smoking status, medication duration, IL-6, exercise habits, and elevated CRP levels. The variance inflation factor (VIF) and tolerance were used for measurement. The results are shown in **Table 2**. The test results indicated that the VIF values of all independent variables were less than 5, and the tolerances were far greater than 0.1, which indicated that there was no serious multicollinearity problem among the independent variables. Therefore, all variables were included in the subsequent logistic regression analysis. The MS was taken as the dependent variable (no=0, yes=1), while age, BIM, smoking (no=0, yes=1), IL-6, medication time, exercise habits (no=0, yes=1), and elevated CRP levels (no=0, yes=1) were used as independent variables. The specific results were shown in **Table 3**. It was found that advanced age (*B* = 0.042; *OR*:1.042; 95% *CI*:1.009, 1.077; *p* = 0.012), high BMI (*B* = 0.446; *OR*:1.562; 95% *CI*:1.341, 1.818; *p*<0.001), smoking habit (*B* = 1.365; *OR*:3.917; 95% *CI*:2.023, 7.586; *p*<0.001), and IL-6 levels (*B* = 4.152; *OR:*63.568, 95% *CI*:17.742, 227.758; *p*<0.001), long medication time (*B* = 0.181; *OR*:1.199; 95% *CI*:1.088, 1.320; *p*<0.001), and elevated CRP levels (*B* = 1.157; *OR*:3.182; 95% *CI*:1.088, 9.307; *p* = 0.035) were risk factors for the development of MS. Moderate exercise habits (*B*=-1.437; *OR*:0.238; 95% *CI* = 0.095; 0.593; *p* = 0.002) was a protective factor for the development of MS.

**Table 2:** Multicollinearity test of influencing factors between the MS group and the non-MS group.

Indicators	VIF	Tolerance
Age	1.057	0.946
BMI	1.135	0.881
Smoking habit	1.410	0.709
IL-6	1.211	0.821
Medication time	1.124	0.890
Exercise habit	1.436	0.697
Elevated CRP levels	1.177	0.850

BMI, body mass index; IL-6, interleukin-6; CRP, C-reactiveprotein.

**Table 3:** The influencing factors of the development of MS was explored by logistic regression analysis.

Indicators	*B*	*SE*	*Wald χ^2^*	OR	95% CI	*P*
Age	0.042	0.017	6.326	1.042	1.009~1.077	0.012
BMI	0.446	0.078	32.990	1.562	1.341~1.818	<0.001
Smoking habit	1.365	0.337	16.398	3.917	2.023~7.586	<0.001
IL-6	4.152	0.651	40.664	63.568	17.742~227.758	<0.001
Medication time	0.181	0.049	13.546	1.199	1.088~1.320	<0.001
Exercise habit	-1.437	0.466	9.491	0.238	0.095~0.593	0.002
Elevated CRP levels	1.157	0.548	4.467	3.182	1.088~9.307	0.035
Constant	-4.892	1.246	15.412	0.008	–	<0.001

BMI, body mass index; IL-6, interleukin-6; CRP, C-reactiveprotein.

### 3.3 Clinical indicators comparison between the intervention group and the control group

The baseline demographic and clinical characteristics of the intervention group and the control group are exhibited in **Table 4**. The results indicated that before the intervention began, there was no statistically significant difference between the two groups in the following indicators (all *p*-values were greater than 0.05): demographic characteristics, including gender, age, BMI, educational level, marital situation; lifestyle and medical history, such as smoking habit, drinking alcohol situation, the family history of MS, number of hospitalizations, age of onset; clinical and laboratory indicators, such as medication time, IL-6, SBP, DBP, exercise habits, sleep disorder situation, depression situation, elevated CRP levels situation; and PANSS scores. These results indicated that the two groups of patients were well comparable at the baseline level, and the differences observed between the groups after the intervention could be reasonably attributed to the effect of the intervention measures.

**Table 4:** Clinical indicators comparison between the intervention group and the control group.

Indicators	Intervention group (*n* = 40)	Control group (*n* = 40)	*t/χ^2^*	*P*
	Mean ± SD/n(%)	Mean ± SD/n(%)		
Gender, n(%)			0.201	0.654
Male	20 (50.00)	22(55.00)		
Female	20 (50.00)	18 (45.00)		
Age, year (mean ± SD)	46.35 ± 8.29	48.67 ± 10.33	-1.107	0.272
BMI, kg/m^2^ (mean ± SD)	27.16 ± 2.34	27.54 ± 2.74	-0.668	0.506
Educational level, n(%)			0.050	0.823
High school and below	21 (52.50)	22 (55.00)		
College degree or above	19 (47.50)	18 (45.00)		
Marital situation, n(%)			0.056	0.813
Married	26 (65.00)	27 (67.50)		
Unmarried	14 (35.00)	13 (32.50)		
Smoking habit, n(%)			0.549	0.459
Yes	27 (67.50)	30 (75.00)		
No	13 (32.50)	10 (25.00)		
Drinking alcohol, n(%)			0.069	0.793
Yes	30 (75.00)	31 (77.50)		
No	10 (25.00)	9 (22.50)		
The family history of MS, n(%)			0.148	0.929
Hypertension	2 (5.00)	3 (7.50)		
Diabetes	4 (10.00)	4 (10.00)		
Hyperlipidemia	3 (7.50)	3 (7.50)		
Number of hospitalizations, nos (mean ± SD)	6.15 ± 1.32	6.39 ± 1.82	-0.677	0.500
Age of onset, year (mean ± SD)	26.24 ± 7.26	27.90 ± 8.70	-0.926	0.357
Medication time, week (mean ± SD)	13.97 ± 2.98	15.17 ± 3.66	-1.607	0.112
IL-6 (ng/L (mean ± SD)	2.41 ± 0.37	2.29 ± 0.27	1.656	0.102
SBP, mm Hg (mean ± SD)	127.35 ± 18.10	126.59 ± 17.08	0.193	0.847
DBP, mm Hg (mean ± SD)	74.92 ± 9.15	75.72 ± 9.59	-0.382	0.704
Exercise habit, n(%)			0.157	0.692
Yes	4 (10.00)	3 (7.50)		
No	36 (90.00)	37 (92.50)		
Sleep disorder, n(%)			0.457	0.499
Yes	19 (47.50)	16 (40.00)		
No	21 (52.50)	24 (60.00)		
Depression, n(%)			0.105	0.745
Yes	5 (12.50)	6 (15.00)		
No	35 (87.50)	34 (85.00)		
Elevated CRP level, n(%)			0.346	0.556
Yes	8 (20.00)	6 (15.00)		
No	32 (80.00)	34 (85.00)		
PANSS scores, point (mean ± SD)	71.98 ± 25.96	74.66 ± 28.96	-0.436	0.664

BMI, body mass index; IL-6, interleukin-6; SBP, systolic blood pressure; DBP, diastolic blood pressure; CRP, C-reactiveprotein; PANSS, positive and negative syndrome scale.

### 3.4 Therapeutic efficacy indicators between the intervention group and the control group

The changes in various therapeutic efficacy indicators between the intervention group and the control group are shown in **Table 5**. Before the DMT intervention, there were no statistically significant differences in the metabolic indicators and PANSS scores between the two groups. After the DMT intervention, the BMI, WC, DBP, FBG, and PANSS scores of the intervention group were all lower than those of the control group. The BMI (*F* = 11.504, *p*<0.001), WC (*F* = 15.753, *p*<0.001), DBP (*F* = 37.308, *p*<0.001) and PANSS scores (*F* = 8.056, *p*<0.001) of the intervention group showed a downward trend as the intervention time increased, while the BMI (*F* = 4.560, *p* = 0.012), WC (*F* = 8.337, *p*<0.001), DBP (*F* = 11.940, *p*<0.001) and PANSS scores (*F* = 8.656, *p*<0.001) of the control group showed an upward trend as the intervention time increased.

**Table 5:** Therapeutic efficacy indicators comparison between the intervention group and the control group.

Indicators	Groups	Before intervention	4 weeks of intervention	8 weeks of intervention	Effect	*F*	*η² (95%CI)*
		Mean ± SD	Mean ± SD	Mean ± SD			
BMI, kg/m^2^	Intervention group(*N* = 40)	27.47 ± 10.27	22.31 ± 9.35	17.15 ± 9.21	Time	1.338	0.017 (0.000-0.047)
	Control group (*N* = 40)	28.23 ± 11.68	30.09 ± 11.74	35.93 ± 12.27	Group	21.188*	0.214 (0.165-0.542)
					Group×Time	63.276*	0.448 (0.363-0.473)
WC, cm	Intervention group(*N* = 40)	87.73 ± 10.21	81.05 ± 9.27	75.02 ± 9.25	Time	0.353	0.005 (0.000-0.018)
	Control group (*N* = 40)	88.95 ± 12.95	94.99 ± 13.27	101.49 ± 14.91	Group	30.029*	0.278 (0.222-0.304)
					Group×Time	17.435*	0.183 (0.169-0.292)
SBP, mm Hg	Intervention group(*N* = 40)	119.56 ± 11.27	118.28 ± 10.19	118.02 ± 12.65	Time	0.001	0.000 (0.000-0.042)
	Control group (*N* = 40)	117.64 ± 13.69	118.81 ± 12.57	119.04 ± 12.29	Group	0.008	0.000 (0.000, 0.046)
					Group×Time	0.535	0.007 (0.000-0.030)
DBP, mm Hg	Intervention group(*N* = 40)	74.98 ± 7.49	67.06 ± 6.13	61.51 ± 7.33	Time	8.432*	0.098 (0.126-0.180)
	Control group (*N* = 40)	73.56 ± 8.13	77.27 ± 8.73	82.86 ± 8.83	Group	25.887*	0.249(0.194-0.375)
					Group×Time	24.868*	0.242 (0.222-0.325)
FBG, mmol/L	Intervention group(*N* = 40)	5.89 ± 0.57	5.43 ± 0.44	5.93 ± 0.71	Time	5.235*	0.063(0.067-0.142)
	Control group (*N* = 40)	6.03 ± 0.74	6.02 ± 0.82	6.23 ± 0.94	Group	18.265*	0.190 (0.155- 0.309)
					Group×Time	3.798	0.046 (0.003-0.062)
TC, mmol/L	Intervention group(*N* = 40)	4.62 ± 0.68	4.53 ± 0.53	4.59 ± 0.71	Time	0.008	0.000 (0.000-0.042)
	Control group (*N* = 40)	4.57 ± 0.73	4.37 ± 0.83	4.58 ± 0.79	Group	0.534	0.007 (0.000-0.059)
					Group×Time	0.483	0.000 (0.000-0.024)
LDL-C, mmol/L	Intervention group(*n* = 40)	3.27 ± 0.51	3.15 ± 0.45	3.29 ± 0.31	Time	2.168	0.027 (0.000-0.053)
	Control group (*n* = 40)	3.07 ± 0.57	2.97 ± 0.65	3.05 ± 0.74	Group	6.859*	0.081 (0.003-0.180)
					Group×Time	0.063	0.001 (0.000-0.005)
TG, mmol/L	Intervention group(*N* = 40)	1.62 ± 0.52	1.56 ± 0.57	1.74 ± 0.62	Time	0.169	0.002 (0.000-0.016)
	Control group (*N* = 40)	1.71 ± 0.73	1.62 ± 0.93	1.68 ± 0.81	Group	0.116	0.001 (0.000-0.010)
			-0		Group×Time	0.468	0.006 (0.000-0.022)
PANSS scores, points	Intervention group(*N* = 40)	73.92 ± 26.34	63.13 ± 22.27	53.14 ± 20.47	Time	0.571	0.007 (0.000-0.030)
	Control group (*N* = 40)	72.72 ± 25.89	84.57 ± 25.57	96.82 ± 26.25	Group	7.429*	0.087 (0.009-0.186)
					Group×Time	4.652*	0.056 (0.003-0.073)

*The repeated measures analysis statistic is significant at the 0.05 level. BMI, body mass index; WC, waist circumference; SBP, systolic blood pressure; DBP, diastolic blood pressure; FBG, fasting blood glucose; TC, total cholesterol; LDL-C, low-density lipoprotein cholesterol; TG, triglyceride; PANSS, positive and negative syndrome scale.

## 4 Discussion

Metabolic dysfunction in patients with schizophrenia is a key clinical concern, and it is of great significance to take effective intervention measures at an early stage. The study’s findings demonstrated that advanced age, smoking habits, high BMI and IL-6 levels, long medication time, and elevated CRP levels could increase the risk of developing MS. Among them, the higher OR values of IL-6 and CRP suggested that they might be the core driving factors for the occurrence of MS in patients with schizophrenia. By detecting the levels of IL-6 and CRP in patients, it is possible to promptly screen for high-risk individuals. However, it was noted that there might be potential overestimation or interference from confounding factors. Therefore, the causal explanation should have been cautious. This study also found that moderate exercise habits could reduce the probability of MS, indicating that appropriate exercise was an important method for treating the development of MS in patients with schizophrenia. The possible reasons for the above phenomena were as follows: (1) As age increases, the incidence of underlying diseases, such as diabetes, hypertension, and hyperlipidemia, rises in the population. This change in pathophysiological basis subsequently heightens the risk of MS (28). (2) Patients with schizophrenia smoked at a rate higher than the general population. Moreover, patients with schizophrenia were more sensitive to nicotine addiction, and their withdrawal reactions after quitting smoking were more severe than those of the general population. Smoking could reduce insulin sensitivity, enhance insulin resistance, and thereby lead to obesity and overweight, increasing the risk of MS (29). (3) Patients with schizophrenia often sit for long periods due to their symptoms. An increase in BMI further reduced their athletic ability, decreased energy consumption, aggravated obesity and metabolic disorders, and thus increased the risk of MS (30). (4) IL-6 could indirectly trigger abnormal activation of insulin receptor function through inflammatory signaling pathways, disrupting the normal phosphorylation process of tyrosine residues in its intracellular domain. This phosphorylation disorder will further interfere with the tyrosine phosphorylation levels of insulin receptor substrates (IRS), weakening its binding ability to downstream phosphatidylinositol 3-kinase (PI3K), and thereby affecting the normal transduction of the PI3K/AKT signaling pathway (8). The decline in the activity of this pathway will reduce the transport of glucose transporters (such as glucose transporter type 4) to the cell membrane, directly leading to a decrease in the ability of muscle and fat tissues to take up and utilize glucose (5). As the sensitivity of peripheral tissues to insulin signaling continues to decline, the body gradually shows manifestations of glucose homeostasis disorders such as elevated fasting blood glucose and abnormal glucose tolerance, ultimately forming a systemic insulin resistance state (31). Moreover, persistent high levels of IL-6 also promoted fat breakdown, release of free fatty acids, and synthesis of very low-density lipoprotein (VLDL) in the liver, further exacerbating dyslipidemia and systemic metabolic disorders, thereby significantly increasing the risk of MS (32, 33). (5) The longer the medication time, the more obvious the cumulative effect of the drug in the body. Especially for second-generation antipsychotic drugs (SGAs) such as clozapine and olanzapine, which could significantly increase the risk of weight, blood sugar, and lipid abnormalities, thereby increasing the risk of MS (9). (6) The elevated level of CRP was not only a sign of chronic inflammation, but was also likely to directly participate in the occurrence of metabolic disorders through multiple mechanisms. On one hand, CRP could induce the expression of acute-phase response proteins in the liver and activate inflammatory signaling pathways such as NF-κB, thereby promoting the release of inflammatory factors such as TNF-α and IL-6, interfering with the downstream signal transduction of insulin receptors, and inducing peripheral tissue insulin resistance (34). On the other hand, CRP could also promote the breakdown of adipose tissue and increase the concentration of free fatty acids in the blood. Excessive free fatty acids can deposit in tissues such as muscles and the liver, further inhibiting the insulin signaling pathway through lipid toxicity effects, while promoting gluconeogenesis in the liver and the synthesis of very low-density lipoproteins, exacerbating glucose and lipid abnormalities (35). The above inflammation and metabolic disorders mutually promote each other, forming a vicious cycle, and jointly drive the development of MS. (7) Exercise habits were a necessary way to strengthen the physical function and life quality of patients with schizophrenia, which could enhance treatment compliance and reduce physical complications (36, 37). Previous studies have shown that those who actively participated in physical exercise to control their weight could promote energy consumption, enhance sugar and lipid metabolism, which was beneficial for preventing MS and delaying the progression of the disease (38).

Based on this, this study proposed DMT intervention treatment and explored the impact of this method on the development of MS in patients with schizophrenia. The results showed that the intervention not only significantly improved the physiological indicators and mental symptoms of the patients, but more importantly, the improvement in some metabolic parameters reached a clinically significant threshold. In terms of metabolic parameters, the BMI and WC of patients in the intervention group showed a statistically significant downward trend, among which the improvement in waist circumference was particularly crucial. According to the authoritative guidelines, such as the International Diabetes Federation (24), WC is a core indicator for assessing MS. A reduction of approximately 3–5 centimeters in WC can be considered a clinically significant minimum important difference (39). The reduction in WC observed in this study exceeded this standard, indicating that the patients had achieved substantial improvement in related metabolic risks, rather than just being statistically significant. Additionally, the DBP of the intervention group decreased with the increase in treatment time; the significant reduction in DBP also had potential clinical value, as even a small reduction in DBP was associated with a reduction in risk of MS. According to earlier research, participants’ anxiety and depression might decrease with DMT treatment (40). The DMT was a body-oriented psychotherapy that could regulate emotions and feelings through some intentional and symbolic movements, meet the lack or deficiency of psychological nutrition, and the satisfaction of emotions and psychology could indirectly improve the blood pressure of patients (41). It promoted physical and mental health, but the specific mechanism still needed further exploration. In terms of mental symptoms, after the intervention group patients were given DMT treatment, PANSS scores showed a downward trend with the extension of treatment time. It is demonstrated that DMT treatment improved the cognitive ability of patients with schizophrenia, positively influenced their behavioral states, alleviated their symptoms, avoided the drawbacks of long-term usage of antipsychotic drugs, reduced the hazard of developing MS in schizophrenia patients, and has a wider applicable population range. The review of relevant foreign studies on improving MS of chronic schizophrenia through exercise intervention further proves this point (42). The above results indicated that after DMT treatment for patients with schizophrenia accompanied by MS, some metabolic indicators and mental state levels had changed, and the effect became more obvious with the extension of treatment time. In conclusion, DMT treatment could exert a synergistic effect in improving both the metabolic indicators and mental symptoms of patients with schizophrenia. Although there was no significant change in SBP, this suggested that the impact of DMT on blood pressure may be specific. However, the overall results indicated that this intervention showed certain clinical potential in key metabolic parameters, providing new ideas for the integrated management of schizophrenia. Future studies can further clarify its mechanism of action and optimize the intervention strategy.

However, the results also revealed that the changes in SBP, FBG, TC, LDL-C, and TG levels after DMT treatment were not significant. The results suggested that DMT treatment had little effect on SBP, FBG, TC, LDL-C, and TG levels. The findings of this study are comparable to those of earlier studies (43, 44). The possible reasons for this outcome could be explored from two aspects: the intervention mechanism and the characteristics of the indicators. First, the DMT was a psychosomatic integration intervention method, which utilized mind-body interconnectedness and non-verbal movement to facilitate the expression of subconscious emotions. Techniques such as mirroring and improvisation activated the mirror neuron system, thereby enhancing emotional release, self-awareness, and social behaviors. As DMT emphasized psychological expression over physical intensity, its low-to-moderate intensity activities resulted in minimal energy expenditure and a limited short-term impact on metabolic indicators like blood glucose and lipids (45). During the treatment process, patients mainly performed coordinated limb movements and creative expression through thematic dance activities, using action metaphors to reconstruct connections with the real world and deeply explore psychological conflicts between themselves and the disease (46). This intervention focus might make DMT more prominent in promoting emotional expression, self-awareness, and social interaction, while having relatively limited effects on biochemical metabolic indicators (47). Second, metabolic indicators such as FBG, TC, LDL-C, TG, and blood pressure were influenced by multiple factors, including genetic background, dietary habits, basal metabolic rate, drug use, and long-term lifestyle (48, 49). These factors often had strong inertia and stability, and it was difficult to achieve significant changes through a single behavioral intervention in the short term (50). Moreover, compared with previous DMT intervention studies (51, 52), the intervention period of this study was relatively short, making it difficult for changes in the above indicators to reach statistical differences.

This study provides a reference for exploring the factors influencing MS in patients with schizophrenia and evaluating the intervention function of DMT treatment. However, this study has the following limitations: First, in the research on the influencing factors of MS, the sample size used was limited, which may result in insufficient statistical power, causing some actual effects or inter-group differences to fail to be effectively detected. Future research should address this by increasing the sample size and enhancing the sensitivity of the statistical tests. Second, this study was a single-center design. All subjects came from the same institution, which may have introduced a selection bias. In the future, multicenter studies should be conducted to enhance the external validity of the research and the reproducibility of the results. Additionally, there may be heterogeneity in the individual responses of this study, which could have obscured the overall effect. Future research can further identify the sources of heterogeneity through methods such as matching key baseline variables (such as demographic characteristics, clinical stage, and types of medication) or stratified analysis, thereby enhancing the interpretability and clinical applicability of the results. Finally, this study lacks long-term continuous observation and follow-up on the intervention effect of DMT, making it difficult to fully reflect the potential long-term effects of DMT. Future research should extend the observation period and enhance the clinical significance and reliability of the results.

In summary, this research has demonstrated that advanced age, smoking habits, high BMI and IL-6 levels, long medication time, and elevated CRP levels could increase the probability of MS, while moderate exercise habits could reduce the probability of MS. Clinical attention should be paid to patients with schizophrenia accompanied by MS. DMT treatment applied to patients with schizophrenia accompanied by MS could effectively improve metabolic indicators, relieve clinical symptoms, and enhance the quality of life.

## 5 Conclusion

Advanced age, smoking habit, high BMI and IL-6 levels, long medication time, and elevated CRP levels were risk factors for the development of MS, while moderate exercise habit was a protective factor. As a promising treatment method, DMT treatment could change the metabolic indicators and mental state levels of patients, such as reducing BMI, WC, DBP, and PANSS scores, to achieve the goals of preventing the occurrence and development of MS and strengthening the physical and mental well-being of patients with schizophrenia.

## Data Availability

The raw data supporting the conclusions of this article will be made available by the authors, without undue reservation.
